# The Use of a Computer Display Exaggerates the Connection Between Education and Approximate Number Ability in Remote Populations

**DOI:** 10.1162/OPMI_a_00016

**Published:** 2017-12-01

**Authors:** Edward Gibson, Julian Jara-Ettinger, Roger Levy, Steven Piantadosi

**Affiliations:** 1 Department of Brain and Cognitive Sciences, MIT; 2 Department of Psychology, Yale University; 3 Department of Brain and Cognitive Sciences, University of Rochester

**Keywords:** number comprehension, cross-culture differences, individual differences

## Abstract

Piazza et al. reported a strong correlation between education and approximate number sense (ANS) acuity in a remote Amazonian population, suggesting that symbolic and nonsymbolic numerical thinking mutually enhance one another over in mathematics instruction. But Piazza et al. ran their task using a computer display, which may have exaggerated the connection between the two tasks, because participants with greater education (and hence better exact numerical abilities) may have been more comfortable with the task. To explore this possibility, we ran an ANS task in a remote population using two presentation methods: (a) a computer interface and (b) physical cards, within participants. If we only analyze the effect of education on ANS as measured by the computer version of the task, we replicate Piazza et al.’s finding. But importantly, the effect of education on the card version of the task is not significant, suggesting that the use of a computer display exaggerates effects. These results highlight the importance of task considerations when working with nonindustrialized cultures, especially those with low education. Furthermore, these results raise doubts about the proposal advanced by Piazza et al. that education enhances the acuity of the approximate number sense.

## INTRODUCTION

In order to understand the universal properties of human thought, there has been a burgeoning interest in cross-cultural research focused on remote, nonindustrialized cultures (Henrich, Heine, & Norenzayan, 2010; Norenzayan & Heine, 2005). However, differences in behavior must always be interpreted with care, as culture often unexpectedly influences performance in ways that complicate interpretation (e.g., Berry, 2002; Cole & Scribner, 1974; Medin, Bennis, & Chandler, 2010). Recently, computer interfaces have gained popularity for collecting behavioral data from remote cultures. A danger in interpreting such data is that the participants may be unfamiliar with the testing devices, leading them to perform less well than they might otherwise. A recent example of a potential overinterpretation of results obtained from an indigenous culture using a computer interface comes in the domain of number cognition.

Piazza, Pica, Izard, Spelke, and Dehaene (2013) used a computerized display to evaluate the ability to estimate approximate quantities (e.g., Dehaene, 2011) in the Munduruku, an indigenous population in the Brazilian Amazon. Piazza et al. reported a strong correlation between education and approximate number sense (ANS) acuity over a small sample of adults (*N* = 38). This result is potentially important because it could mean—as Piazza et al. speculate—that “symbolic and nonsymbolic numerical thinking mutually enhance one another over the course of mathematics instruction” (p. 1037). For example, practice with arithmetic might afford a learner the opportunity to calibrate and sharpen their approximate number judgments.

One possible confound, however, is that participants with less education were simply less comfortable with the computer displays, potentially leading to worse performance based solely on their comfort with the testing situation. Piazza et al. attempt to control for this confound by showing that participants were matched on their ability to perform a separate task on a computer display—choosing the larger of two discs—but participants were near ceiling on this task (mean accuracy = 95%), suggesting that this task was too simple to reliably differentiate among individuals. Thus, it is still possible that education may simply predict comfort with a computer display in this indigenous population, rather than participants’ ability in an approximate number task.

To investigate the role of computer displays in number cognition in a population that has little familiarity with computers, we worked with the Tsimane’, a native Amazonian group living in the lowlands of Bolivia (Huanca, 2008). The Tsimane’ live in small groups, hunt, and farm (to a limited extent) for subsistence. Unlike people from industrialized cultures, many Tsimane’ adults have never attended school, and those that have attended often begin school at a later age than individuals in industrialized countries, and they often leave school earlier. Hence their education level is highly variable across the population.

We constructed the present experiment to test whether possible discomfort with the computer presentation would manifest itself in the measurement of ANS acuity, much like effects of task comfort on success that have been observed in U.S. children (Odic, Hock, & Halberda, 2014). For our purposes, such a baseline shift in performance would prevent “fair” comparison of acuity levels across cultures; more generally, a variable influence of task might preclude comparison across any two populations, including adults and children. Even more problematically, interactions between the task effect and education would lead to spurious (or exaggerated) education effects in correlations (as in Piazza et al., 2013): When only computerized displays were used, it would appear as though education improved ANS acuity, when in fact increased education might just allow participants to be comfortable in the testing paradigm.^1^

## EXPERIMENT

One hundred and forty-five adults (mean age: 36.8 years; *SD*: 16.3 years; range: 17–77 years) were recruited from six Tsimane’ communities near the town of San Borja in the Bolivian Amazon, in collaboration with the Centro Boliviano de Investigación y de Desarrollo Socio Integral (CBIDSI), which provided interpreters, logistical coordination, and expertise in Tsimane’ culture.

### Methods

Participants first completed a short demographic survey, including reporting the highest number of years of education they had achieved (a whole number between 0 and 16), their age, gender, Spanish proficiency, and household size. Tsimane’ education consists of classroom work in the village, with the local teacher (usually the most educated person in the village). Children learn the basics of arithmetic, reading, writing, Spanish language, and training in needed skills for village living, such as how to build houses.

**Figure 1.**
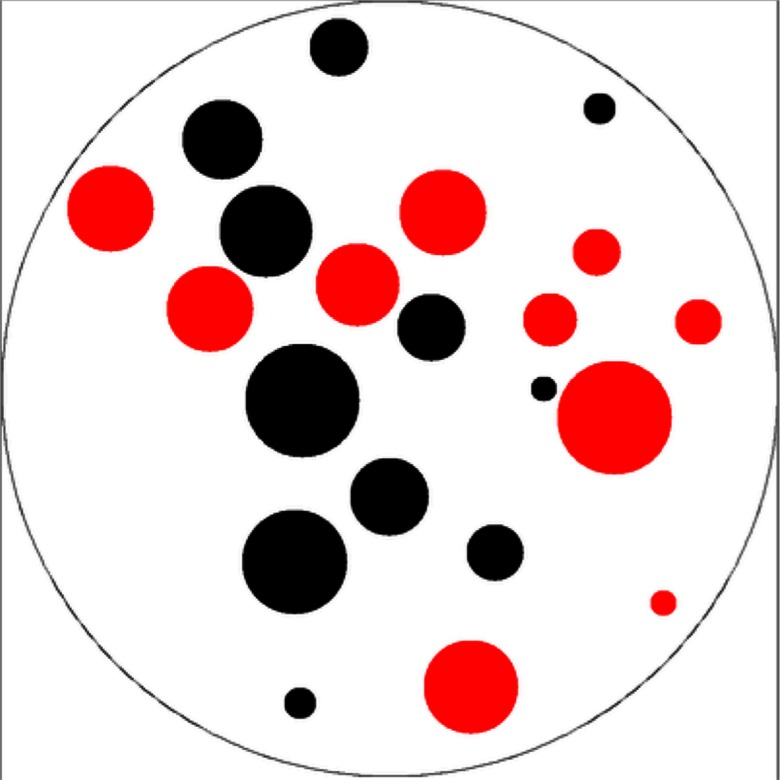
An example stimulus consisting of black and red dots of varying sizes, intermixed inside a disc.

Our main task consisted of two parts, performed in a random order for each participant. Area-controlled, intermixed dot stimuli were presented in two different ways to each participant: (a) via a touchscreen laptop computer and (b) via laminated cards that were presented by the experimenters. The stimuli consisted of black and red dots of varying sizes, intermixed inside a disc (see Figure 1). For each version of the task, participants were asked to report whether there were more black or red dots in the display. The sets of black and red dots were matched for the size of the biggest and smallest dots in each set. The sets varied in ratios among the following ratios, going from least to most complex to discriminate: 1:3, 1:2, 2:3, 3:4, 4:5, 5:6, 6:7, 7:8, 8:9, 9:10, 10:11, and 11:12. In order to minimize spurious differences among the perceivable ratios, we kept the total number of dots as close to a total of 20 as possible, given these ratios (i.e., 5:15, 7:14, 8:12, 9:12, 8:10, 10:12, 12:14, 7:8, 8:9, 9:10, 10:11, and 11:12). There were eight versions of each of the ratios, each with four trials where “red” was the correct answer, and four where “black” was correct.

Participants were instructed to touch a red square below the presentation disc if there were more red dots or a black square if there were more black dots. These squares were on the screen of the touchscreen computer version, or on a laminated card in the card version. Stimuli remained in front of the participant until they touched one of the squares. In the cards version of the task, the correct answer was printed on the card in a coded form. The experimenter would put his thumb over this code when placing the card in front of the participant, so that neither he nor the participant could see it until the trial was complete. This way, the experimenter could not provide cues to the participants about correct answers.

Participants were trained on eight practice trials consisting of dots in a 1:3 ratio. If they made any mistakes, the experimenter would explain the task again, and then they would repeat the set of eight practice trials, in a different random order. Participants were given at most three attempts to complete the practice trials correctly. Four participants failed this criterion and no further data were collected for them. Once a participant finished one version of the task (computer or cards), they would start the other version (cards or computer). We report data from the 141 participants who succeeded in the practice trials (78 who did the cards version first; 63 who did the computer version first).

In the test part of each task, participants performed 30 total trials, starting at the 1:2 ratio, and using a two-up, one-down staircase design, such that if they got two answers in a row correct at one level, they moved up a level of difficulty. If, however, they got an answer wrong, then they were moved down a level. Participants who got trials incorrect at the 1:2 ratio continued at the 1:2 ratio. The eight trials per level were randomized/shuffled before each participant.

### Analysis

All subjects’ behavior on each task was characterized by fitting a Weber fraction (W) to their entire set of responses. The Weber fraction W indexes the amount of variance in participants’ ANS number representations, such that a smaller Weber fraction indicates better performance and sharper Gaussian curves. We use Piantadosi’s (2016) method for fitting W, which is closely related to the maximum likelihood fitting used widely in the field (e.g., Halberda, Mazzocco, & Feigenson, 2008), but introduces a weak prior bias (for small W) in order to combat the problem that high W are difficult to distinguish statistically. The small bias decreases the variance of each subjects’ estimated W, while introducing a negligible influence on the mean of the estimate, leading to quantifiably better estimates.^2^ We treat all subjects’ fit Ws as point estimates of their acuity in each ANS task. We predict Log W from our dependent features.

### Results

**Figure 2.**
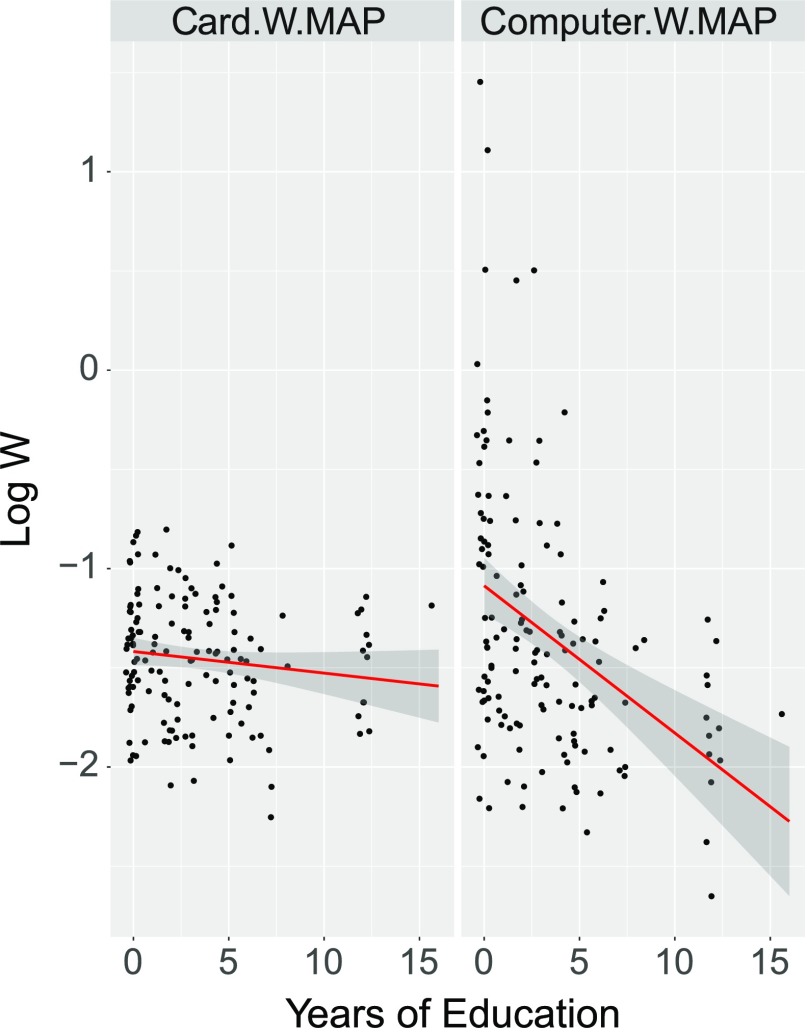
**Plot of the relationship between years of Tsimane’ education and Log(W) for the two versions of the task: Cards vs. Computer.** As shown in Table 1, there is a reliable interaction between Tsimane’ education and task, such that there is a strong correlation between education and Log W for the computer version of the task on the right, but nothing reliable in the card version on the left (see Table 2).^4^

Data and analyses are available at http://osf.io/ctaj4 (Gibson, 2017). A statistical evaluation of the relationship between Education and Log W for the sum-coded card and computer versions of the task is provided in Tables 1 and 2. Figure 2 shows the relationship between years of education and Log W for the two versions of the task. As is visually apparent in the figure, there is a reliable interaction between Tsimane’ education and task, such that there is a strong correlation between education and Log W for the computer version of the task on the right, but much less so in the card version on the left. These correlations are presented in Table 2.

**Table 1.** A linear mixed effects regression (including a by-subject random intercept to account for repeated within-subjects measurements) predicting Log W from Tsimane’ education level and task (computer vs. card version).

**AIC**	**BIC**	**logLik**	**deviance**	**df.resid**
401.8	423.7	−194.9	389.8	276

Scaled residuals:				
Min	1Q	Median	3Q	Max
−2.1824	−0.6418	−0.0226	0.4943	4.9975

Random effects:				
Groups	Name	Variance	*SD*	
Subject	(Intercept)	0.02481	0.1575	
Residual		0.20978	0.4580	
Number of obs: 282, groups: subject, 141				

Fixed effects:				
	Estimate	*SE*	*t* value	
(Intercept)	−1.252289	0.041399	−30.249	
Education	−0.042551	0.008400	−5.066	
task1	−0.165655	0.037229	−4.450	
Education:task1	0.031667	0.007554	4.192	

Correlation of fixed effects:				
	(Intr)	Eductn	task1	
Education	−0.681			
task1	0.000	0.000	−0.681	

Note: summary(lmer(W_value_lg ∼ Education * task + (1 | subject), REML=F, data=gathered_d)) Linear mixed model fit by maximum likelihood [’lmerMod’] Formula: W_value_lg ∼ Education * task + (1 | subject)

**Table 2.** Linear regressions predicting Log W from Tsimane’ education level for the computer task (highly significant) followed by the card task (nonsignificant).

Residuals:				
Min	1Q	Median	3Q	Max
−1.1209	−0.4343	−0.1016	0.3188	2.5396

Coefficients:				
	Estimate	*SE*	*t* value	Pr(>| t |)
(Intercept)	−1.08663	0.07125	−15.251	<2e–16 ***
Education	−0.07422	0.01446	−5.134	9.39e–07 *

Residuals:				
Min	1Q	Median	3Q	Max
−0.75909	−0.20395	0.02276	0.23466	0.63624

Coefficients:				
	Estimate	*SE*	*t* value	Pr(>| t |)
(Intercept)	−1.417943	0.034819	−40.723	<2e–16 ***
Education	−0.010884	0.007065	−1.541	0.126

Note: lm(formula = W_value_lg ∼ Education, data = just_comp) ^†^*p* < .1. *p < .05. **p < .01. ***p < .001.

**Figure 3.**
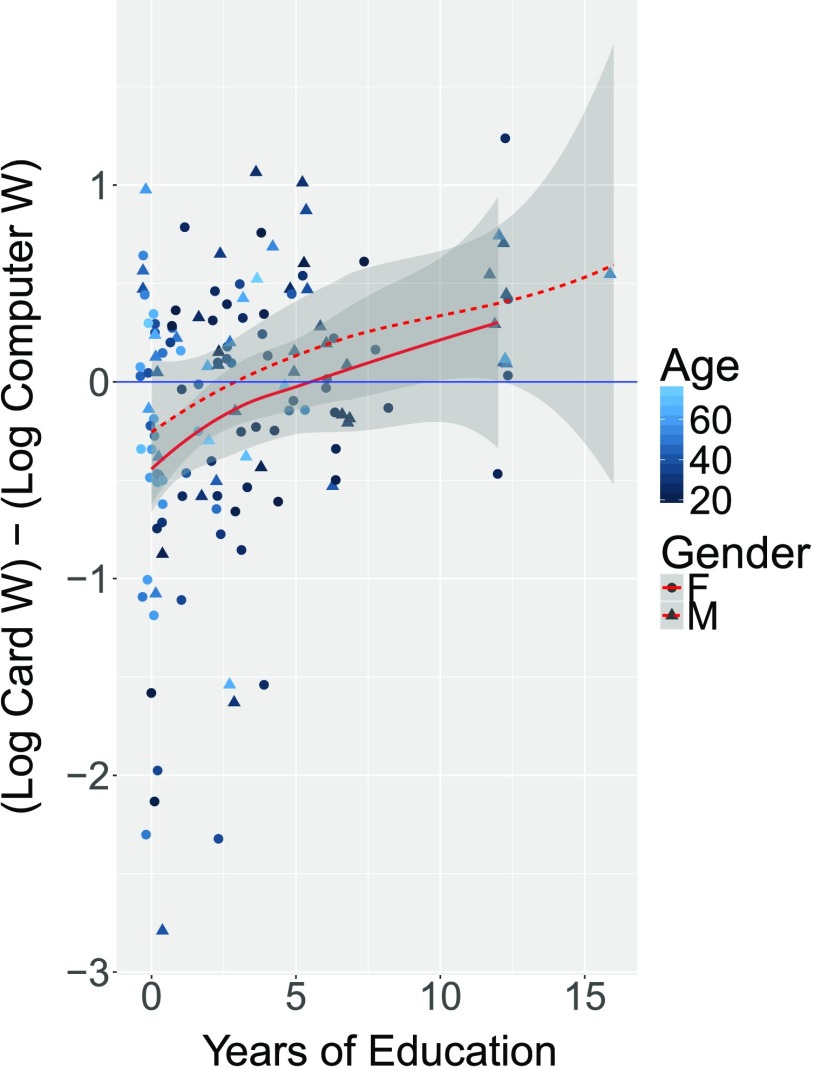
**Difference score (Log Cards W minus Log Computer W) as a function of education for males and females separately (which don’t differ significantly).** A smoothed nonparametric fit (loess) is shown for each, with 95% confidence bands. This figure demonstrates that for low education, Log Card W is less than Log Computer W (negative values on this plot), meaning that participants perform less well on computer tasks. However, the positive trend of the red average line indicates that the effect disappears and potentially reverses for high education. In addition, this figure reveals no obvious trends with respect to age and the difference score, but one can see that high-education participants tend to be younger and male, reflecting current Tsimane’ demographics.

Figure 3 shows another visualization of these data, giving the difference score (Cards minus Computer) as a function of education. A smoothed nonparametric fit (loess) is shown for each of males and females, with 95% confidence bands. This figure demonstrates that for low education, Log Card W is less than Log Computer W, meaning that participants perform better on the card task. However, the positive trend of the average line indicates that the effect disappears with high education. In addition, this figure reveals no obvious trends with respect to age and the difference score, but one can see that high education participants tend to be younger and male, reflecting current Tsimane’ demographics.

To assess statistical significance, we computed a linear regression predicting the difference in Log W (Cards) minus Log W (Computers) from demographic and task factors (see Table 3). The resulting fit suggests that adults with no education perform significantly worse when the task is administered on a computer interface, as the intercept is significantly less than zero. The regression reveals a significant effect of education such that the difference between the tasks vanishes with increasing education. The magnitude of the coefficients accords with Figure 3: The tasks do not differ after approximately 5 or 6 years of education (.287 / .051 = 5.6 years). The regression also included a (sum-coded) predictor for which task was run first within participants: when the computer version was run first, the difference between the computer and cards version was larger. In addition, the regression reveals no effect of (standardized) age, but a marginal effect of gender (sum-coded), such that the difference between the computer and cards version was slightly larger for women than men.^3^ When we omit participants with more than 10 years of education, we find very similar statistical trends as in the analyses reported in the table: All reliable effects are also reliable here. Thus it is not the 13 Tsimane’ participants with 12+ years of education that are driving the observed effects. Finally, the regression in Table 4 shows that Tsimane’ education is largely predicted by age and gender: More educated Tsimane’ participants tend to be young and male.

**Table 3.** A linear regression predicting the difference in Log W (Cards minus Computers) from demographic and task factors.

**Residuals:**				
Min	1Q	Median	3Q	Max
−2.4145	−0.3365	0.1191	0.4094	1.3290

**Coefficients:**				
	Estimate	*SE*	*t* value	Pr( >|t|)
(Intercept)	−0.28733	0.08092	−3.551	0.000528 ***
Education	0.05106	0.01663	3.071	0.002579 **
Comp.First.sum	−0.38043	0.10809	−3.519	0.000589 ***
scale(Age)	0.01967	0.05797	0.339	0.734911
Gender1	−0.09847	0.05915	−1.665	0.098286 ^†^

Note: lm(formula = CardsMinusComputers.lg ∼ Education + Comp.First.sum + scale(Age) + Gender, data = d). ^†^*p* < .1. *p < .05. **p < .01. ***p < .001.

**Table 4.** A linear regression showing that Tsimane’ education is largely predicted by age and gender: More educated Tsimane’ people tend to be young and male. (This accounts for the gender and age effects that are visible in Figure 3.

**Residuals:**				
Min	1Q	Median	3Q	Max
−5.6181	−2.0344	−0.3949	1.2314	11.9871

**Coefficients:**				
	Estimate	*SE*	*t* value	Pr( >|t|)
(Intercept)	3.5706	0.2849	12.532	< 2e–16 ***
scale(Age)	−1.2454	0.2818	−4.420	1.99e–05 ***
Gender1	−1.2057	0.2849	−4.232	4.22e–05 ***
scale(Age):Gender1	−0.4277	0.2818	−1.518	0.131

Note: lm(formula = Education ∼ scale(Age) * Gender, data = d). ^†^*p* < .1. *p < .05. **p < .01. ***p < .001.

## DISCUSSION

Our results demonstrate that participants with lower education levels performed worse on the task with the computer display than with the card display, whereas participants with higher education levels did just as well on the card or computer versions of the task. These results emphasize the importance of task comfort and understanding, particularly when working with populations that are unfamiliar with experimental psychology and behavioral paradigms.

The fact that task performance is influenced by education level suggests that, if we had not noticed the potential confound with task, we might have found a spurious education effect. Indeed, as seen in Table 2, if we only analyze the effect of education on W, as measured by the computer version of the task, the effect is statistically significant, even though the effect of education on the card version of the task is not. The variable effect of education within the Tsimane’ population on these task factors might therefore have led us to conclude that education strongly influenced ANS if we had run only the computer version. Of course, the influence of task does not show that there is *no* education effect, only that if task is not controlled, we cannot be sure. This is a plausible alternative explanation for the findings of Piazza et al. (2013), discussed above. Though it is plausible that education influences ANS in these populations (we find a small nonsignificant tendency in this direction), detailed controls for task are required to rule out alternative explanations.

A comparison of the slope of the effect in our computer task—about .034 W / year (note that the regression in Table 2 is computed over Log W, not W)—to the slope of the effect in Piazza et al. (2013)—about .25 W / year—suggests that the Piazza et al. effect is much larger, even on the matched computer task. But this comparison assumes that the education years are matched. Alternatively, it is possible that the education in the Munduruku is more organized than in the Tsimane’, leading to a larger effect each year. Note that the effect is miniscule for the Tsimane’ cards task—.003 W / year—and this is not significant in the regression.

There are several plausible explanations for the strong education effect on task that we observed, due to factors that correlate with educational level. First, it is not the case that experience with computers could explain the observed differences, because almost none of the participants had ever seen a computer or computer tablet before, independent of their educational level, according to their own self-reports. One possible source for the education effect on task is more experience with technology more generally, such as radios, TV screens, and phones. People with more education are more likely to travel to the local Bolivian (Spanish) towns, where there is access to technology, such as a television in the town square. Another possibility is that people with greater education might have better developed cognitive control (Brod, Bunge, & Shing, 2017; Burrage et al., 2008; Morrison, Smith, & Dow-Ehrensberger, 1995; Roebers, Röthlisberger, Cimeli, Michel, & Neuenschwander, 2011) so that they can better ignore irrelevant aspects of the testing situation, such as the novel computer presentation. People with lower education might have a harder time focusing on the relevant aspects of the task in the novel situation. Thus, removing computer interfaces from cross-cultural studies would not address the current concerns.

These results also have important ramifications beyond cross-cultural research: anywhere where familiarity with technology may covary with another dimension, such as age, socio economic class, or gender. In such cases, participants might be unfamiliar with computers, and researchers should therefore be careful to show that their participants don’t behave differently depending on how the task is administered.

Our results show that one should be careful when designing tasks with participants who are not used to cognitive research, and careful about interpreting results from studies on remote populations, especially if a study purports to show performance that is different from a population with industrialized education. If the remote group performs similarly to industrialized nation participants, then the remote group understood the task as well as the industrialized participants (e.g., Dehaene, Izard, Pica, & Spelke, 2006, 2008; Izard, Pica, Dehaene, Hinchey, & Spelke, 2011; Izard, Pica, Spelke, & Dehaene, 2011; McCrink, Spelke, Dehaene, & Pica, 2012; Pica, Jackson, Blake, & Troje, 2011; Pica, Lemer, Izard, & Dehaene, 2004). But when they perform relatively poorly, this may not reflect a genuine cognitive difference between groups.

Perhaps most importantly, our results suggest that the effect of education on ANS that Piazza et al. (2013) had observed in the Munduruku may be much weaker—if it exists at all—than suggested by Piazza et al.’s study. Future work will be needed to see if there is a reliable correlation between education and ANS that is unconfounded by task.

## AUTHOR CONTRIBUTIONS

EG, JJE, and STP designed the study. EG, JJE, and RL carried out the experiment. STP did the analyses. EG and STP drafted the manuscript. JJE and RL provided critical feedback on the manuscript, and all authors contributed to the final draft of the manuscript.

## ACKNOWLEDGMENTS

We thank Ricardo Godoy and Tomas Huanca for logistical help. Dino Nate Añez, Robertina Nate Añez, and Salomon Hiza Nate helped with translating and running the task. We thank Evelina Fedorenko and Rachel Ryskin for comments on earlier drafts of this paper. Research reported in this publication was supported by National Science Foundation Grant 1022684 from the Research and Evaluation on Education in Science and Engineering (REESE) program to EG. The project was also supported by the Eunice Kennedy Shriver National Institute of Child Health & Human Development of the National Institutes of Health under Award Number F32HD070544 to STP. The content is solely the responsibility of the authors and does not necessarily represent the official views of the National Institutes of Health.

## Notes

^1^ Although there is a large literature on computer vs. paper tasks in the education literature, this literature predominantly involves reading, such as the TOEFL (Test of English as a Foreign Language) or SAT tasks (for a review, see Noyes & Garland, 2008). The results from this literature suggest that there is either a slight benefit for doing tasks on paper rather than computer, across all levels of participants, or no benefit either way (which seems to be the tendency in the more recent literature, possibly due to [a] better computer screens for task presentation and [b] people being more used to working on computer screens in recent years). Whereas this literature is potentially related to our research question, our ANS task involves no reading whatsoever. Furthermore, we were most interested in whether there are differences according to education level. But we are not aware of any literature on computer versus paper tasks reporting interactions with education.^2^ The statistical patterns are very similar when we used standard maximum likelihood fits: Any effect that we report as significant using Piantadosi’s methods is also significant using maximum likelihood fits.^3^ In another analysis, we investigated a potential interaction between education and task order, but this effect was nonsignificant (*p* = .12), so we left this interaction term out of the presented analysis. Although not significant, the direction of this interaction was such that lower education participants had a larger difference score.^4^ The regressions in Table 2 are similar if we predict W instead of Log W. In particular, for the computer task the relationship is significant (beta = −.034, *t* = −3.13, *p* = .002), but for the card task the relationship is not (beta = −.0029, *t* = −1.67, *p* = .098).
